# A Bioanalytical Liquid Chromatography Tandem Mass Spectrometry Approach for the Quantification of a Novel Antisense Oligonucleotide Designed for Parkinson’s Disease: A Rat Brain Biodistribution Study

**DOI:** 10.1021/acsptsci.4c00698

**Published:** 2025-02-05

**Authors:** Anastasia Palaiologou, Marianna Naki, Marina Pantazopoulou, Fedon-Giasin Kattan, Leonidas Stefanis, Epaminondas Doxakis, Constantin Tamvakopoulos

**Affiliations:** † Center of Clinical Research, Experimental Surgery and Translational Research, Division of Pharmacology-Pharmacotechnology, Biomedical Research Foundation, Academy of Athens, Soranou Ephessiou Street 4, Athens GR-11527, Greece; ‡ Center of Basic Research, Biomedical Research Foundation, Academy of Athens, 11527 Athens, Greece; § Department of Physiology, National and Kapodistrian University of Athens (NKUA), 11527 Athens, Greece; ∥ Center of Clinical Research, Experimental Surgery and Translational Research, Biomedical Research Foundation, Academy of Athens, Soranou Ephessiou Street 4, Athens GR-11527, Greece

**Keywords:** LC-MS/MS, antisense oligonucleotide (ASO), brain distribution, Parkinson’s

## Abstract

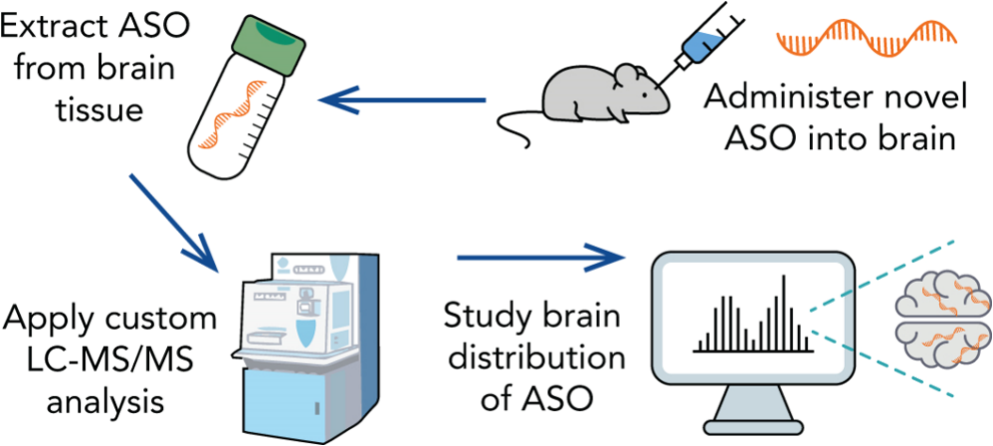

Antisense oligonucleotides (ASOs) represent a unique category of therapeutics targeting disease-related RNAs. Since this new therapeutic category emerged, the immediate need to analyze ASOs in clinically relevant biological matrices has led to several methodologies, such as ligand binding assays and imaging techniques. To overcome issues in specificity and provide exact quantitative data for ASOs, a new LC-MS/MS method was developed to analyze in brain tissue a novel 4–10–4 gapmer ASO with the potential for treating Parkinson’s disease with phosphorothioated backbone and 2′-O-(2-methoxyethyl) modifications. The sample pretreatment protocol to extract the ASO from brain tissue employed solid phase extraction (SPE) and protein digestion. The LC-MS/MS method was fully optimized, validated and applied to quantify the target ASO in brain tissue samples following an *in vivo* brain distribution study. The method has a Lower Limit Of Quantification of 1 ng/mg and was applied to the study’s samples, demonstrating satisfactory sensitivity and providing valuable information about the ASO’s distribution in different brain regions over 45 days.

Advances in next-generation sequencing technologies have identified the genetic roots of many common diseases.^1^ DNA and mRNA, protein precursors, are attractive therapeutic targets due to Watson–Crick base pairing,^2^ laying the foundation for antisense oligonucleotides (ASOs). ASOs, small nucleic acid sequences (13–30 bases), are complementary to RNA targets representing a unique category of therapeutics. ASOs can change the expression levels or the splicing defects of a protein by targeting disease-related RNAs and modify their splicing, translation or abundance. ASOs uniquely address a broad spectrum of rare and genetic diseases.^2^

The first successful use of an ASO for *in vivo* gene regulation was in 1978 when Rous sarcoma viral replication was inhibited in chicken embryos.^3^ Two decades later Fomiversen was approved in the US and Europe for treating retinitis cytomegalovirus in AIDS patients.^4^ Currently, 12 ASO drugs are approved and over 100 are in different phases of clinical development.^5−7^ Since ASOs emerged as a therapeutic category, the need to analyze them in various clinically relevant biological matrices has led to new methodologies. Liquid chromatography-tandem mass spectrometry (LC-MS/MS) has emerged as a highly selective technique for ASO analysis, able to differentiate and quantify intact ASOs and their metabolites across a wide dynamic range, making it valuable for biodistribution assessments.^5^ Analytical approaches for ASOs are challenging due to their unique physicochemical properties. The characteristic phosphate backbone of ASOs makes them highly polar, thus making it difficult to retain them in a conventional reverse-phase chromatographic column. To overcome this issue, ion-pairing (IP) chromatography is employed, adding positively charged alkylamines in the mobile phase to form ion pairs with the negatively charged ASOs. This combination drastically improves retention; consequently, ASOs are primarily analyzed with IP chromatography.^5^ Other approaches, such as Hydrophilic Interaction Liquid Chromatography (HILIC), have also been reported.^8^

While several approaches have been described to monitor ASOs distribution in the Central Nervous System (CNS), they present limitations. Single photon emission computed tomography (SPECT) nuclear imaging provides valuable ASOs distribution data without animal sacrifice but cannot quantify ASOs, limiting SPECT to qualitative data, precluding the establishment of pharmacokinetic profiles, that lead to concentration-related conclusions.^9^ Immunoassays lack specificity and cannot distinguish full-length ASOs from metabolites or synthetic impurities, often resulting in overestimated analyte concentrations. They also exhibit poor tissue sensitivity due to the extensive dilutions required.^10^ A number of studies quantitated ASOs via enzyme-linked immunosorbent assays (ELISA) to monitor brain regions after IT administration in clinical studies^11^ or spinal cord distribution in NHP.^12^ ASO concentrations were also measured via capillary gel electrophoresis.^12,13^

LC-MS/MS affords superior specificity and exact quantification of ASOs in CNS distribution studies but, oligonucleotide analysis in tissues using mass spectrometry remains a challenge, with a limited number of publications.^5,14−16^ The most studied tissues are the liver and kidneys since those organs play a crucial role in drug metabolism and excretion. Tissue-related studies refer to the metabolic stability of different ASOs^17^ or metabolite profiling and pharmacokinetics.^16^ Structural differences affecting ASOS *in vivo* behavior were also studied.^15^ Focusing on the brain, it remains largely understudied using LC-MS/MS methodologies. In addition, to the best of our knowledge, only a limited number of studies provide validation data.^10^ A representative study regarding ASO analysis in brain tissue uses hybridization with biotinylated sense-strand oligonucleotides coupled to streptavidin magnetic beads to extract ASOs from tissues.^10^ A major drawback, clearly stated for such approaches, is that capture probes with 100% complementary sequences are used for each ASO, resulting in a different probe needed for every studied sequence, making this an expensive approach. This approach is also employed to study drug unbound fraction of three different ASOs (MOE-gapmers) in the brain.^18^ Another study conducted to compare extraction protocols, primarily in plasma, used 20 different ASOs, with 3 of them also analyzed in brain tissue. However, the Lower Limit of Quantification (LLOQ) was not reported for this specific matrix.^19^

This study presents the development, validation and application of a bioanalytical LC-MS/MS method for quantifying ASOs in rat brain tissue. A sample preparation protocol was optimized to be adaptable to different sequences or tissues. The monitored ASO is a novel 4–10–4 gapmer with a phosphorothioated backbone and 2′-O-(2-methoxyethyl) modifications designed to target the α-synuclein (SNCA) gene in Parkinson’s disease. The resulting data provides valuable information about the novel ASO’s biodistribution, contributing to its preclinical pharmacokinetic profile. This work demonstrates the critical role of such analytical approaches in developing more effective therapeutics.

## Materials and Methods

### Materials

The oligonucleotides used in this study were synthesized by GenScript Biotech (Rijswijk, Netherlands) (Table 1). Solid Phase Extraction (SPE), Clarity OTX 96-well plates (100 mg/well) and Clarity OTX Lysis-Loading buffer were obtained from Phenomenex (Aschaffenburg, DE). The HPLC column (Clarity 2.6u Oligo-XT 100A 100 × 2.1 mm) and precolumn (SecurityGuard ULTRA Cartridges Clarity Oligo-XT) were also obtained from Phenomenex (Aschaffenburg, DE).

**Table 1:** ASO 3.0 and Internal Standard (IS)

name	MW (kDa)	sequence length	sequence	chemistry^a^
ASO 3.0	6.4	18	**5′-CAACTTCTGAACAACAGC-3′**	4–10–4 MOE-gapmer, PS
IS	7.1	20	**5′-TAAGTATTGTGCTGCTTCCT-3′**	5–10–5 MOE-gapmer, PS

a MOE: 2′-O-(2-methoxyethyl)-oligoribonucleotides, PS: phosphorothioate backbone.

### Instrumentation and LC-MS/MS Conditions

IP agent, dibutylamine (DBA), and 1, 1, 1, 3, 3, 3-hexafluoro-isopropanol (HFIP), were added in the mobile phase. The total analysis time was 12.5 min with baseline resolution for analyte and IS, at a flow rate of 0.5 mL/min in 55 °C column temperature.

A SCIEX QTRAP 5500+ mass spectrometer equipped with Turbo Ion Spray source (SCIEX, Concord, ON, Canada) was used for the analysis. The mass spectrometer was operated in the Multiple Reaction Monitoring (MRM) mode and in the negative ionization mode. For ASO 3.0 two transitions were selected and monitored. 705 → 95 *m*/*z* was used as the quantifier ion and 794 → 95 *m*/*z* as the qualifier ion. The quantifier/qualifier ion ratio was monitored in all analytical runs and used for analyte identification. IS transition was 791 → 95 *m*/*z*. Detailed conditions are presented in the Supporting Information.

### Solution and Standard Preparation

ASOs (ASO 3.0, IS) were received as dry powders and dissolved in saline at 200 mg/mL. A 1 mg/mL stock solution prepared in H_2_O was stored at −20 °C, from which all working stocks were prepared in H_2_O with appropriate dilutions. Working stocks were spiked into brain tissue homogenates to produce calibration standards at 1, 2, 4, 8, 10, 20, 40, 80, 160 ng/mg and Quality Control solutions (QCs) at 8, 40 ng/mg. Detailed steps about solutions preparation, blank tissue harvesting and calibration standards and QCs preparation can be found in the Supporting Information.

### Sample Preparation

**Figure 1:**
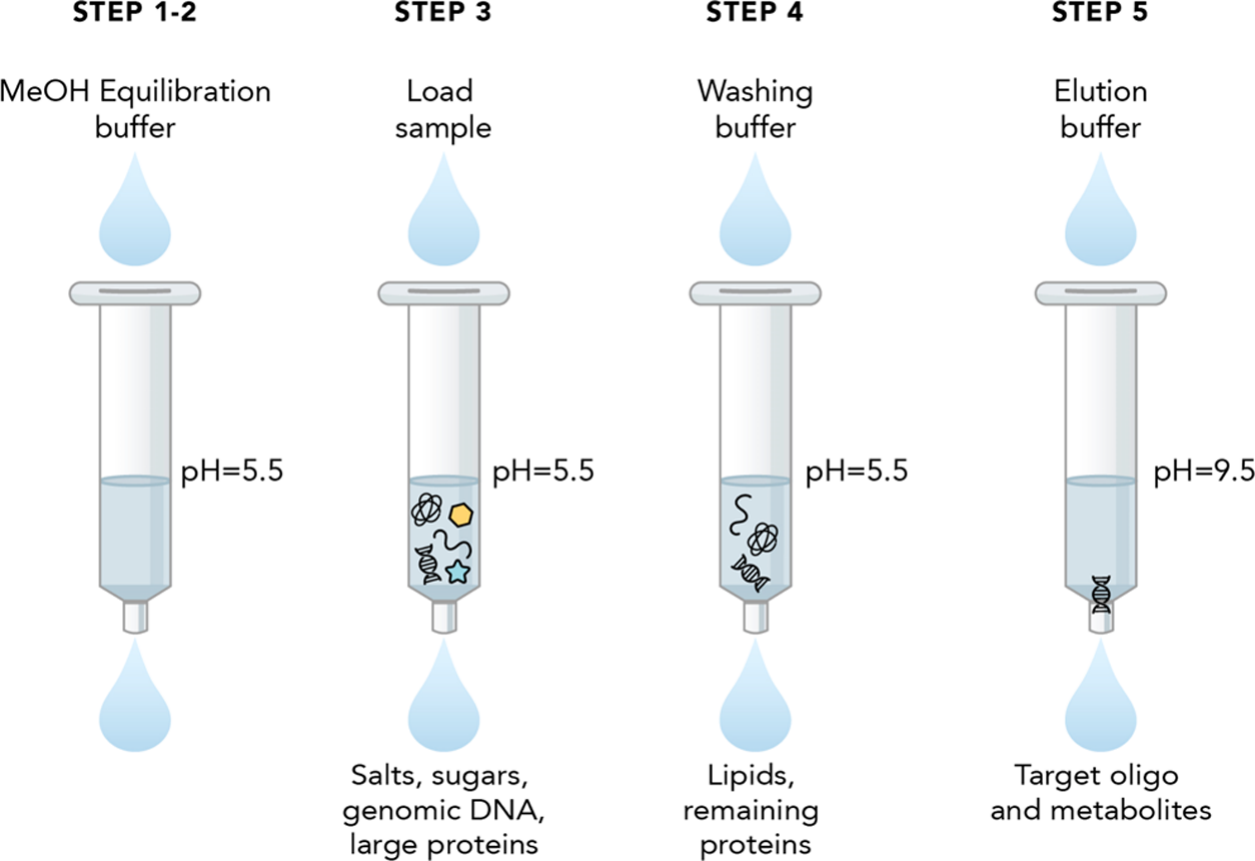
SPE steps followed for sample cleanup.

A volume of 10 μL of tissue homogenate was digested by Proteinase K at 55 °C for 3 h. Solid Phase Extraction (SPE) followed, appling digested tissue homogenates diluted in Clarity Lysis-Loading buffer into Clarity OTX extraction plates. Samples were loaded (pH = 5.5), washed and finally ASOs were eluted (pH = 9.5). Solvents were evaporated at 55 °C and the dry residue was reconstituted in mobile phase to be analyzed. SPE steps are presented in Figure 1 and detailed sample preparation steps are described in Supporting Information.

### Method Validation

The developed method was validated according to the ICH M10 on bioanalytical method validation guideline by the European Medicines Agency.^20^ The validation process included accuracy, precision, linearity, selectivity, matrix effect, and recovery assessments.

### Animal Study—Collection of Rat Brain Sections

The ASO 3.0 was administered to rats (Sprague–Dawley rats, Tg(SNCA)BAC) by intracerebroventricular injection (ICV). The ASO 3.0 was prepared in saline at 200 mg/mL and administered at 3 mg/animal. The rats were sacrificed, and samples were collected at 2, 7, and 45 days after administration. Three brain regions were selected for analysis: cerebellum, cortex, and midbrain (dorsal and ventral), to get a preliminary assessment of the ASO 3.0 distribution in the brain. The samples were snap-frozen in dry ice immediately after dissection and stored at −80 °C. Animal experimentation was approved by the competent Veterinary Service of the Prefecture of Athens under Presidential Decree 56/2013, harmonizing the national legislation to the European Directive 2010/63 for protecting animals used for scientific purposes.

## Results and Discussion

### LC-MS/MS Optimization

During the development of the LC-MS/MS method, the focus was primarily on optimizing the mobile phase additives. An IP reagent was essential for ASOs retention, and an acidic modifier was necessary to adjust the mobile phase pH. As previously reported, implementing IP chromatography is complex, as different IP agents result into varying performance patterns that depend on the oligonucleotide sequence.^21^ Consequently, optimizing the IP agent utilized in a LC-MS/MS methodology must be explicitly evaluated for the analyte of interest during method development.

Lower MS signal intensity for oligonucleotides was observed in the absence of the IP agent compared to when an IP agent was added. Alkylamine reagents have been widely used in the LC-MS analysis of ASOs since they assist with the desorption of ions into the gas phase during ionization, favor charge-state reduction, and decrease cation adduction, overall resulting in enhanced MS signal intensity.^22^ Typically, volatile bases like amines, have been selected to achieve a sharp charge-state distribution in the MS spectrum, minimizing clustering due to adduct formation and maximizing signal intensity.^23^

**Figure 2:**
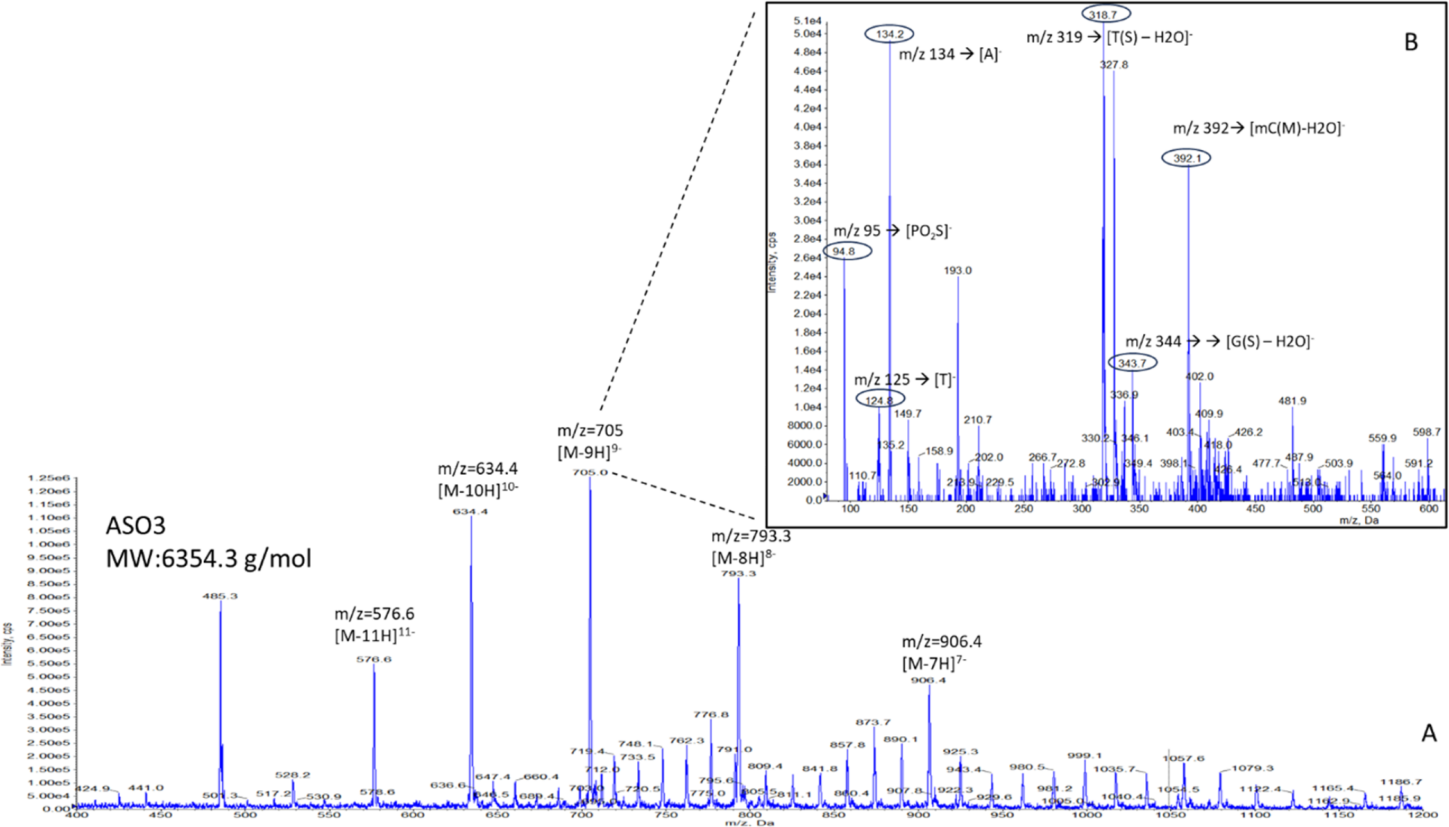
Mass spectra. (A) multiply charged spectra of the ASO 3.0 precursor ions. (B) MS/MS fragments of *m*/*z* 705. m: methyl, (S): phosphorothioate, (M): 2′-O-methoxyethyl plus phosphorothioate.

Five different IP agents were tested in direct infusion experiments, examining the adduct formation and the charge-state profile of ASO 3.0. All IP agents were alkylamines used at low (5 mM) concentrations, preferred in early method development to protect the MS system, as alkylamines have been reported to cause MS contamination, resulting in ion suppression when switching from negative to positive ionization.^21^ N,N-diisopropylethylamine (DIEA), and triethylamine (ΤEA) were rejected due to extensive adduct formation. N,N-dimethylcyclohexylamine (DMCHA) resulted in reduced adduct formation but DBA and hexylamine (HA) produced the sharpest charge-state envelopes. DBA was ultimately selected due to higher signal intensity. Precursor ion 705 *m*/*z* representing the (M-9H)^9–^ state and its product ions are presented in Figure 2. After establishing the optimum IP reagent, other MS-related parameters were optimized. As previously reported,^10^ all selected precursor ions when further fragmented produced similar product ions, characteristic of nucleic acids with the same structural modifications. Indicatively, fragment *m*/*z* 95 represents the phosphodiester backbone (P_2_S^–^) while nucleotide and nucleoside-originated fragments were also observed (*m*/*z* 125 → thymine, *m*/*z* 134 → adenine, *m*/*z* 319 → thymine phosphate, *m*/*z* 344 → guanosine phosphate) and PS nucleotides such as *m*/*z* 392→ 2-MOE methyl cytosine minus a water molecule.

The LC-MS/MS system was further protected from the IP reagents used for LC analysis by diverting the LC flow to waste before and after the elution of the analytes. The curtain gas flow was also increased from the typical setting of 20 to 30 (arbitrary units) to mitigate the previously reported performance deterioration associated with using the reagents mentioned above.^14^

After MS-related parameters and IP reagent (DBA) were established, the mobile phase composition was optimized and an acidic modifier was added. Given that oligonucleotides are ionized via electrospray ionization (ESI) in negative ion mode, acidic modifiers play a dual role: carrying excess negative charge and neutralizing the solution’s pH.^22^ HFIP has been used for this purpose since its introduction to the field, since various mobile phase additives were tested in the past but mostly failed to successfully provide a balance between HPLC separation and MS signal suppression.^24^ HFIP acts by adjusting the solution pH while simultaneously enhancing the MS signal intensity of oligonucleotides.^22^ HFIP is reported to be more volatile than other acids used for pH adjustment, potentially aiding the desolvation of the solvent droplet during the ESI process. In addition, HFIP is only partially ionized at pH 7–8.3 (p*K*_a_ 8.25), thus minimally interfering with the oligonucleotide ionization.^23^ As HFIP evaporates from the droplet, the pH reportedly becomes more basic, from 7 to 10, due to the remaining oligonucleotide-amine basic complex, resulting in increased sensitivity of the LC-MS/MS system.^25^

**Figure 3:**
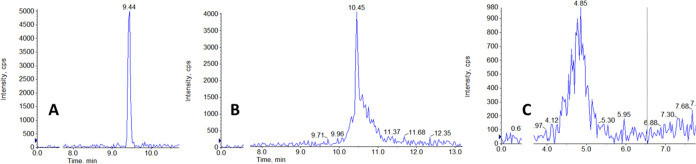
Chromatograms of ASO 3.0 obtained while optimizing mobile phase additives. (A) 20 mM HFIP—15 mM DBA. (B) 40 mM HFIP—15 mM DBA. (C) 20 mM HFIP—30 mM DBA. The ASO 3.0 concentration was maintained constant.

**Figure 4:**
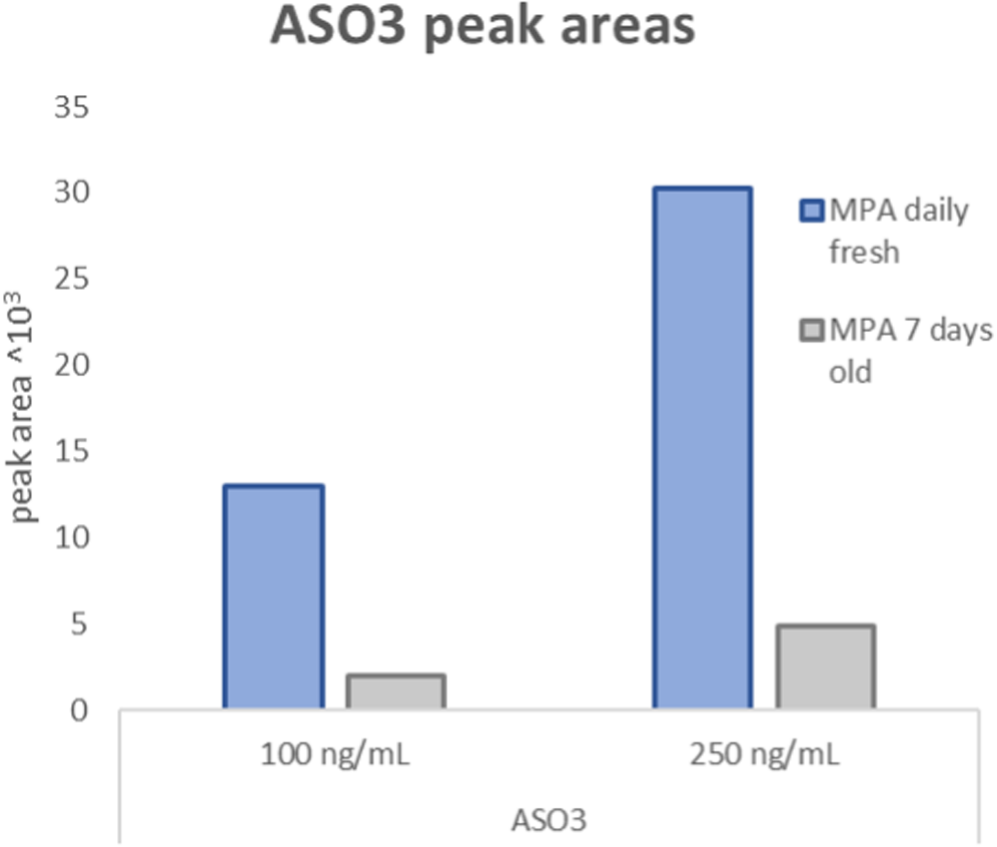
Comparison of ASO 3.0 at two concentrations analyzed with freshly prepared versus 7-day-old mobile phase.

DBA (15 mM) and HFIP (20 mM) were added in both aqueous and organic mobile phases, and a test gradient was applied, which resulted in a sharp peak shape for ASO 3.0 (Figure 3a). Further investigation was conducted for both mobile phase additives to verify that the applied concentrations were sufficient for the purposes of this analysis. HFIP was increased to 40 mM (Figure 3b) and DBA to 30 mM (Figure 3c). In both cases, the peak shape deteriorated compared to the sharp peak shown in Figure 3a. Therefore, these concentrations (15 mM DBA, 20 mM HFIP) were selected and the gradient program was further optimized to achieve baseline separation for the two analytes (ASO 3.0, IS). Additionally, as signal deterioration for nonfreshly prepared mobile phase was previously reported,^10^ this issue was investigated. ASO 3.0 solutions (100 ng/mL, 250 ng/mL) were analyzed using 7-day-old versus freshly prepared mobile phase. Figure 4 shows lower peak areas for quantifier and qualifier ions with the 7-day-old mobile phase. This observation led to the decision to prepare mobile phase fresh on the day of analysis.

### Sample Preparation

Protein precipitation (PP), enzyme digestion, liquid–liquid extraction (LLE) and solid phase extraction (SPE) have been extensively described for preparation and cleanup of samples for ASOs analysis.^26^ Additionally, magnetic bead approaches have recently been reported as a novel and promising approach for sample preparation.^10^ PP is suggested as the simplest way to remove proteins from biological fluids. However, for ASOs, PP is characterized by low recovery values (5–10%) due to the strong protein binding of ASOs, which results in coprecipitation of the analyte during sample preparation.^27^

For tissue sample preparation, PP had been employed for various organs (heart, kidneys, liver, lungs, spleen); however, the recovery of the method was not addressed.^14^ LLE had been reported primarily for liver and kidney tissues^15,25^ with high recoveries mentioned in one case and appears to be an affordable and relatively simple approach. However, since the solvents used for the extraction are mainly phenol and chloroform, these protocols were avoided, and more environmentally friendly approaches were pursued. Extraction procedures using SPE have been increasingly preferred in recent years,^17,19^ with reported recoveries exceeding 80%. The technique presents several advantages, as it can be automated, miniaturized, and coupled online with chromatography.^28^ Finally, hybridization approaches are highly selective and yield nearly 100% recoveries, but they increase analysis costs as custom probes are required for each ASO sequence.^10^

Considering the above, SPE was chosen for sample preparation. Specifically, Clarity OTX cartridges were selected, and the extraction was based on a generic protocol for tissue samples, suggested by the manufacturer, and further optimized for the specific goals of this study. These cartridges employ a weak anion exchange (WAX) mechanism to extract oligonucleotides, utilizing MS-compatible reagents. In summary, the cartridges are equilibrated at pH 5.5 to retain the negatively charged oligonucleotides. After several washing steps to clear the sample of unwanted components, such as lipids and protein residues, the oligonucleotides are finally eluted by neutralizing the cartridges at pH 9.5. The Lysis-Loading buffer in which the sample is loaded into the cartridge, disrupts the protein binding of oligonucleotides, facilitating their extraction. As previously reported, the elution buffer was modified to include the antioxidant TCEP.^19,29^ TCEP is an MS-compatible antioxidant for sulfur compounds, which is suggested to prevent the oxidation of the phosphorothioate backbone of the oligonucleotide during the evaporation step. TCEP was added to the elution buffer, the evaporation procedure was tested and yielded a recovery of 108% ± 12, indicating that this long-lasting step (3h) at a high temperature (55 °C) does not compromise analyte stability.

**Figure 5:**
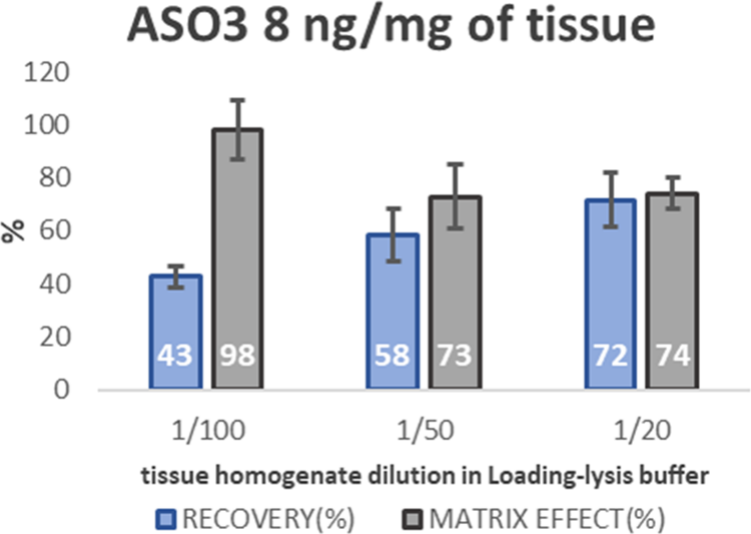
Recovery and matrix factor calculated for different dilutions of tissue homogenate in loading-lysis buffer.

To optimize the tissue homogenate/buffer ratio for posthomogenization sample loading into the cartridges, various dilutions of the homogenate were tested as tissue/buffer ratios 1:10 or even 1:20 (w/v) improve the recovery of oligonucleotides.^19^ Following this rationale, various dilutions of tissue homogenate (1/100, 1/50, 1/20) in Lysis-Loading buffer were tested and evaluated, monitoring recovery and matrix effects. Results are presented in Figure 5.

The matrix effects, presented in Figure 5, indicate the enhancement or suppression of the analyte signal by coeluting matrix components. To quantify this effect, the matrix factor was calculated as the ratio of analyte peak area in samples spiked with ASO 3.0 after the extraction procedure versus the analyte peak area of neat solutions. Recovery, also presented in Figure 5, expresses the percentage of the analyte extracted from the matrix. It was calculated as the ratio of the analyte peak area in samples spiked with ASO 3.0 before the extraction procedure versus the analyte peak area in samples spiked with ASO 3.0 after the extraction procedure.

Dilution of tissue homogenate in Lysis-Loading buffer (1/100) resulted in a matrix factor of 98% ± 11, indicating minimal matrix effects, but yielded a lower recovery (43% ± 4). Dilution of tissue homogenate (1/50) slightly increased recovery to 58% ± 10 and produced a matrix factor of 73% ± 12. Dilution of tissue homogenate (1/20) improved recovery to 72% ± 10, while the matrix factor remained at 74% ± 6.

The dilution of 1/20 was ultimately selected as optimum since it produced the best recovery (72% ± 10) while indicating a relatively low matrix effect, with a matrix factor calculated at 74% ± 6. The recovery and matrix factor were also calculated at a high concentration of ASO 3.0 (40 ng/mg). Results are presented in Table 2.

**Table 2:** ASO 3.0 Recovery and Matrix Factor at Two Concentration Levels

	recovery (%)	matrix effect (%)
8 ng/mg	68 ± 9	81 ± 10
40 ng/mg	80 ± 15	61 ± 7

Protein binding is common to all drugs. For ASOs in the brain, it has been reported that the drug unbound fraction in rat brain tissue is 7-fold lower compared to rat plasma, likely due to the brain’s higher protein content.^18^ Therefore, combining the SPE approach with another sample preparation procedure to release the ASO 3.0 from proteins before further cleanup was deemed necessary. Concerning tissue samples, Proteinase K digestion is often combined with SPE.^17^ It was previously mentioned that Proteinase K digestion does not influence recovery in brain tissue samples as in liver or kidney tissues.^19^ Indeed, a similar recovery (72% ± 10 without Proteinase K digestion versus 68% ± 9 with proteinase K digestion) was demonstrated when adding the Proteinase K digestion step before SPE, with a slight improvement in the matrix factor (74% ± 6 vs 81% ± 10 when adding the Proteinase K digestion step). Following these results and aiming to adopt this extra sample preparation step to ensure that samples are optimally free of proteins before applying SPE, a recovery experiment was performed to evaluate only the Proteinase K digestion step. A recovery of 95% ± 9 was calculated, suggesting that adding the extra step of Proteinase K digestion will not significantly affect the overall recovery of the protocol but would result in cleaner extracts.

### Selectivity and Sensitivity

**Figure 6:**
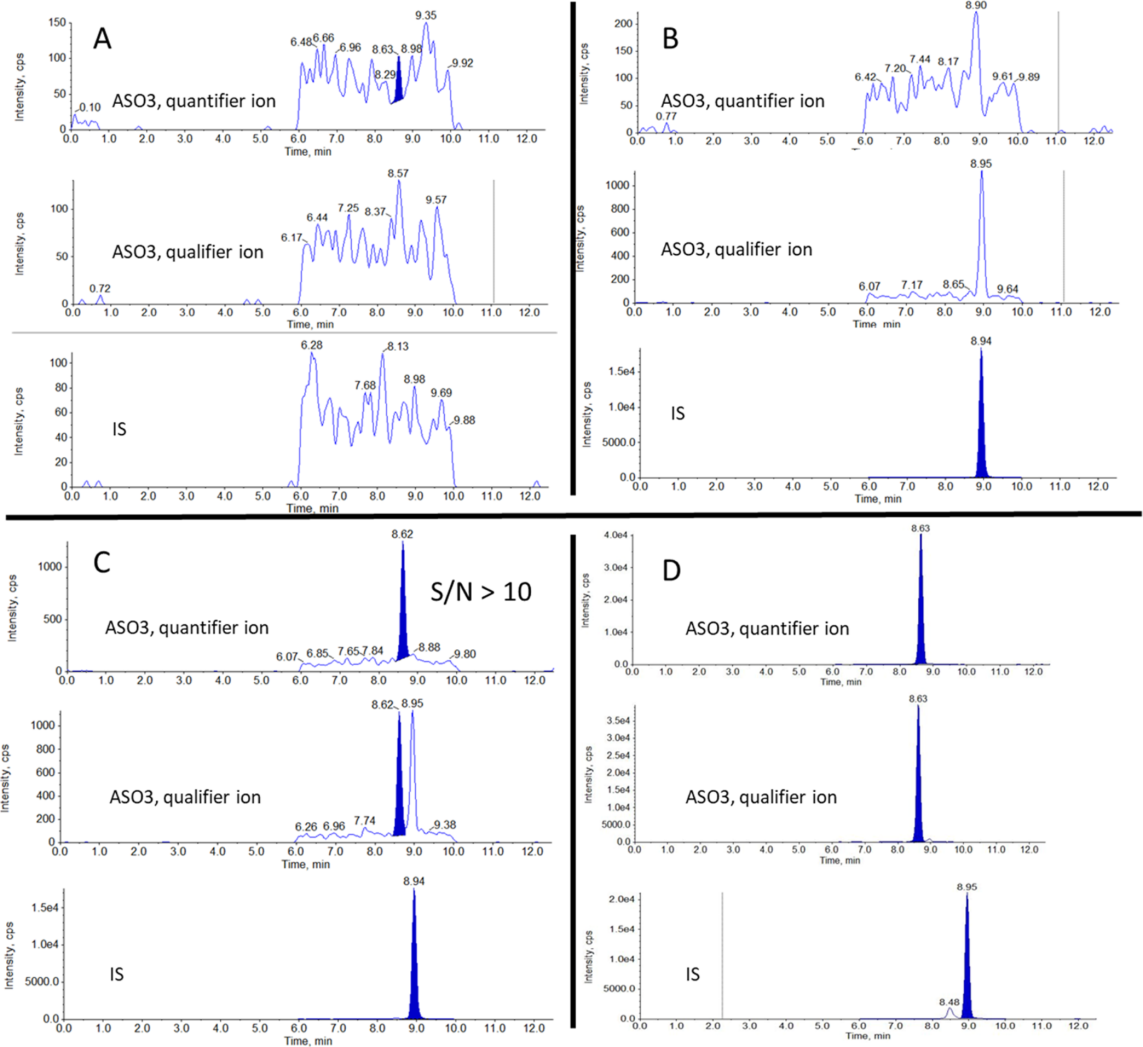
Representative chromatograms showing from top to bottom ASO 3.0 quantifier ion, ASO 3.0 qualifier ion, IS for (A) blank sample, (B) zero sample, (C) LLOQ 1 ng/mg, and (D) 160 ng/mg.

The optimized LC-MS/MS method was used to analyze tissue samples, producing chromatograms with satisfactory selectivity and sensitivity for the ASO 3.0 and the IS. Figure 6 shows representative chromatograms of (A) a double blank, (B) a blank with IS, (C) LLOQ (1 ng/mg), and (D) high concentration (160 ng/mg) for ASO 3.0 and IS. The double blank appears clear of peaks at the respective retention times of ASO 3.0 (8.6 min) and the IS (8.9 min), with no interferences from the matrix.

When adding IS in the zero blank, a small peak at the retention time of IS is observed in one of the ASO 3.0 monitored transitions. Such crosstalk is commonly observed in oligonucleotide analysis due to the frequent use of close or overlapping Q1 precursor ions and the choice of the less-specific product ion *m*/*z* 95 because of its high abundance.^10^ In the presented study, the transition of ASO 3.0 where the crosstalk was observed was designated as the qualifier ion, used solely to confirm the analyte of interest and not for quantification. Furthermore, baseline separation was achieved for the two molecules, ensuring that this slight crosstalk would not be a problem for the analysis, as it appears at a different retention time compared to ASO 3.0. However, it is essential to remember that in contrast to the common practice in analytics, where isotopically labeled internal standards are preferred, with oligonucleotide analysis, this is not a viable choice since less-specific MRM transitions are usually monitored. Considering this, a sequence of similar lengths for which baseline separation can be achieved is the best choice. In this context, the advantage is that similar sequences are unlikely to be misidentified as the target analyte while effectively accounting for variations due to evaporation and instrument performance.^10^

The LLOQ chromatogram demonstrates a signal-to-noise ratio above 10, as shown in Figure 6C, which is the commonly accepted thresshold.^30^ The baseline separation previously discussed can be verified in this chromatogram, ensuring no ASO 3.0-IS confusion can occur.

As an example, clinical data from a phase 2, open-label, dose-escalation study of Nusinersen^11^ shows that for brain autopsy tissues collected from subjects 12 to 78 days after drug dosing, Nusinersen concentrations ranged from 3.7 to 31.8 ng/mg. This case indicates that our method is well-suited for application in clinical settings.

### Linearity

A calibration curve was produced against which all samples were quantified. The calibration range was 1–160 ng/mg. An analyte/IS peak area ratio was used for quantitation and plotted against the nominal concentration for seven calibration standards, producing an equation for quantitation of unknown samples, with typical *r*^2^ > 0.999. A linear regression model was applied with a weighting factor of 1/*x*.

### Accuracy and Precision

Accuracy and precision were assessed both throughout a single run (within-run) and in three separate runs over 2 days (between-run), using spiked tissue homogenate QC samples at two different concentration levels (8 and 40 ng/mg, *n* = 4). Accuracy was calculated as [%AR = (measured concentration/nominal concentration) × 100] and precision as the percent coefficient of variation (%CV). Results are presented in Table 3. %AR was within ±15% of the nominal concentration and %CV did not exceed ±15%.

**Table 3:** Results for Within-Run and Between-Run Accuracy and Precision of ASO 3.0 at Low and High QCs

low QC (8 ng/mg)							high QC (40 ng/mg)						
**within-run**							**within-run**						
	no.	measd concn (ng/mg)	average (*n* = 4)	SD	precision (%CV)	accuracy (%)		no.	measd concn (ng/mg)	average (*n* = 4)	SD	precision (%CV)	accuracy (%)
**day 1**	1	8.7	8.2	0.5	**5.5**	**97**–**109**	**day 1**	1	38.7	36.7	1.9	**5.2**	**85**–**97**
	2	7.7						2	37.2				
	3	8.0						3	34.1				
	4	8.5						4	36.9				
**day 2**	1	7.2	7.2	0.2	**2.2**	**87**–**91**	**day 2**	1	39.0	38.2	1.8	**4.6**	**89**–**99**
	2	7.2						2	38.4				
	3	7.3						3	35.6				
	4	6.9						4	39.6				
**day 3**	1	8.0	8.6	0.4	**4.1**	**100**–**114**	**day 3**	1	39.6	39.1	1.4	**3.6**	**93**–**100**
	2	9.1						2	37.0				
	3	8.6						3	39.6				
	4	8.3						4	40.1				
			**between-run**							**between-run**			
			average (*n* = 12)	SD	precision (%CV)	accuracy (%)				average (*n* = 12)	SD	precision (%CV)	accuracy (%)
			8	0.7	**8.7**	**87**–**114**				37	1.8	**4.9**	**85**–**100**

### Analysis of Rat Brain Regions following in Vivo Study

The ASO 3.0 was administered to rats via ICV injection. Due to size and negative charge, ASOs cannot permeate the blood-brain barrier. Therefore, delivery typically occurs via intracerebroventricular (ICV) injections or lumbar intrathecal injections (IT),^31^ which enables ASO distribution throughout the spinal cord and brain parenchyma.^12,32^ Brain regions (cerebellum, cortex, midbrain) were collected at three different time points (2, 7, 45 days). The hypothalamus was also collected at 45 days. All samples were processed and analyzed based on the described method. Samples were quantified based on calibration curves constructed in blank brain tissue and results are presented in Table 4. A limitation of our study was that only two rats were used for each time point, resulting in concentrations with high variability at 2 and 7 days. Such variability suggests that more dosed animals are needed to draw definitive conclusions, something that is beyond the scope of the presented work. These results though, align with previous studies demonstrating that single-stranded phosphorothioate-modified and 2′-MOE-modified ASOs injected into the CNS rapidly distribute throughout the spinal cord and into most brain regions.^11−13,33,34^ 2′-MOE-modified ASOs present a tissue half-life in CNS tissues between 3 weeks and 6 months in mice and nonhuman primates (NHP). The effects on gene expression from single-stranded phosphorothioate ASOs can last 6 weeks to 6 months, depending on the chemical design of the ASO. This fact also supports the infrequent administration of these drugs.^33,34^ After ICV administration, the highest drug concentrations are found near the injection site in the tissue surrounding the ventricle. This was also the case in this study, where higher concentrations at 2 and 7 days were measured in the cortex where the injection site was located. These patterns of distribution have been observed in mice, rats, cynomolgus and Rhesus monkeys, dogs, pigs, and humans.^35^

**Table 4:** Results of ASO 3.0 Measured in Brain Tissue Samples Following ICV Administration

	time point (days)	2 days (*n* = 2)	7 days (*n* = 2)	45 days (*n* = 2)
		concentration (ng/mg of tissue)^a^		
tissue	cerebellum	7.3 (13.5, 1.0)	12.7 (6.5, 18.8)	6.2 (6.7, 5.8)
	cortex	31.1 (20.2, 42.0)	34.3 (42.0, 26.5)	6.8 (7.6, 6.1)
	midbrain	5.9 (10.8, 0.9)	12.6 (7.1, 18.1)	2.3 (2.9, 1.7)
	hypothalamus^b^			6.2 ± 2.0

a Presented as average (measured values from two different animals),

b *n* = 3.

## Conclusions

This study presents the development, validation, and application of a new bioanalytical LC-MS/MS method. The method was applied to quantify ASO 3.0 in brain tissue samples. A preliminary brain distribution study in rats was conducted. ASO 3.0 was administered via ICV injection, and concentration levels over a specific period were quantified. ASO 3.0 was successfully detected and quantified in all three brain regions analyzed at three different time points, up to 45 days. This is the first reported method in brain tissue using a sample preparation approach of combined protein digestion with Proteinase K and SPE, with reported LLOQ at 1 ng/mg, applied to a rat brain distribution study. The method requires only 5 mg of sample tissue for analysis and demonstrates selectivity, sensitivity, linearity, accuracy, and precision for ASO 3.0 quantification in brain tissue.

Overall, compared to other studies regarding ASOs distribution in the CNS that use techniques with lower specificity, nonvalidated procedures, or unable to quantify the ASOs directly, the present study demonstrates a uniquely comprehensive approach that combines a validated method with its application in an *in vivo* brain distribution study. The approach for sample preparation is generic and can also be employed to extract ASOs from other tissues or different sequences from brain tissue. The resulting data provided valuable information about the biodistribution of the novel ASO 3.0, contributing to establishing its preclinical pharmacokinetic profile.

This innovative study was applied to brain tissue, which is highly understudied when evaluating ASOs. As this emerging category of therapeutic agents primarily targets the CNS, developing methodologies that precisely quantify the drug in target brain regions is of great importance for creating new, effective therapeutic agents. Recent reductions in MS instrument costs and improvements in user-friendly software have increased the accessibility of this technology. Anticipated advances in data analysis tools are expected to further enhance it. Combined with advancements in instrumentation improving sensitivity, these approaches have become a more attractive option. Consequently, the availability of validated methods and generic sample preparation protocols, such as those presented in this manuscript, is expected to make this approach more accessible to users in both industry and academia.

## Supporting Information Available

LC-MS/MS conditions, solution preparation, sample preparation (PDF)
