# Supplementary material for: A Bioanalytical Liquid Chromatography Tandem Mass Spectrometry Approach for the Quantification of a Novel Antisense Oligonucleotide Designed for Parkinson’s Disease: A Rat Brain Biodistribution Study
Source: ACS Pharmacol Transl Sci. 2025 Feb 5;8(2):592–601. doi: 10.1021/acsptsci.4c00698 (PMC11833717; doi:10.1021/acsptsci.4c00698)
Supplement: Supplementary file 1 — pt4c00698_si_001.pdf [file pt4c00698_si_001.pdf]

A bioanalytical Liquid Chromatography tandem Mass Spectrometry approach for the quantification of a novel antisense oligonucleotide designed for Parkinson’s disease. A rat brain biodistribution study.

Anastasia Palaiologou[1], Marianna Naki[2,3], Marina Pantazopoulou[4], Fedon-Giasin Kattan[2], Leonidas Stefanis[4], Epaminondas Doxakis*[2], Constantin Tamvakopoulos*[1]

[1]Center of Clinical Research, Experimental Surgery and Translational Research, Division of Pharmacology-Pharmacotechnology, Biomedical Research Foundation, Academy of Athens, Soranou Ephessiou Street 4, Athens GR-11527, Greece.
[2]Center of Basic Research, Biomedical Research Foundation, Academy of Athens, 11527 Athens, Greece.
[3]Department of Physiology, National and Kapodistrian University of Athens (NKUA), 11527 Athens, Greece.
[4]Center of Clinical Research, Experimental Surgery and Translational Research, Biomedical Research Foundation, Academy of Athens, Soranou Ephessiou Street 4, Athens GR-11527, Greece.

* Correspondence: Constantin Tamvakopoulos, Email: ctamvakop@bioacademy.gr, Epaminondas Doxakis, Email: edoxakis@bioacademy.gr

MATERIALS & METHODS

Other Materials

Water (H_2O), methanol (MeOH), acetonitrile (ACN) LC-MS grade, and tetrahydrofuran (THF) were purchased from Fisher Scientific (Loughborough, UK). All amines, 1, 1, 1, 3, 3, 3-hexafluoro-isopropanol (HFIP), EDTA, ammonium acetate, ammonium bicarbonate, acetic acid,mammonium hydroxide and DNA Lobind tubes were purchased from Merck KGaA (Darmstadt, DE).

Instrumentation and LC-MS/MS conditions

An HPLC system (Exion LC AB Sciex) with a temperature-controlled column compartment and an autosampler was used for the chromatographic separation of ASOs. A Clarity 2.6 μm Oligo-XT 100A (Phenomenex Aschaffenburg, Germany) 100 x 2.1 mm column was selected and combined with a pre-column (SecurityGuard ULTRA Cartridges Clarity Oligo-XT, Phenomenex Aschaffenburg, Germany) to protect and expand the column's lifetime. The optimized chromatographic conditions are presented in Table S1.

Table S1. LC-related conditions.

**Mobile Phase (MP)**	MP A: 90% H_2O-10% MeOH, 20mM HFIP, 15mM DBA<br>MP B: 10% H_2O-90% MeOH, 20mM HFIP, 15mM DBA
**Injection volume**	10 μL
**Autosampler temperature**	10 °C
**Flow rate**	0.5 mL/min
**Column temperature**	55 °C
**Gradient program (%B, min)**	(15, 4.00), (40, 8.00), (40, 9.50), (15, 9.51), (15, 12.50)
**Needle wash**	H_2O/MeOH (50/50 v/v)
**Analysis time**	12.5 min
HFIP: 1, 1, 1, 3, 3, 3-hexafluoro-isopropanol, DBA: dibutylamine	

A SCIEX QTRAP 5500+ mass spectrometer equipped with Turbo Ion Spray source (SCIEX, Concord, ON, Canada) was used for the analysis of ASOs. Table S2 presents the optimal conditions and monitored transitions.

Table S2. MS-related conditions.

**Source temperature**		550 °C			
**Ion spray voltage**		-4500 V			
**Gas settings (arbitrary units)**		CUR: 30, CAD: 8, GS1: 50, GS2: 40			
**Analyte**	**Transition**	**Dwell (msec)**	**DP (V)**	**CE (V)**	**CXP (V)**
**ASO3**	705→95(quantifier ion)	50	-119	-125	-17
	794→95 (qualifier ion)	50	-133	-125	-38
**IS**	791→95	50	-136	-150	-11
CUR: curtain gas, CAD: collision gas (Nitrogen), GS1: nebulizing gas, GS2: heater gas, DP: declustering potential, CE: collision energy, CXP: collision cell exit potential					

AB Sciex Analyst software (version 1.7.1) was used for all data acquisition and processing. Calibration curves were generated by plotting peak area ratios (analyte/IS) against nominal concentrations and applying linear regression.

Standard and Quality Control solutions

ASOs (ASO3, Internal Standard-IS) were received as dry powders and dissolved in saline at 200 mg/mL. Solutions were stored at -80 ^{o}C. A 1 mg/mL stock solution prepared in H_2O was stored at -20°C, from which all working stocks were prepared in H_2O with appropriate dilutions at concentrations of 0.5-1-2-4-5-10-20-40-80 µg/mL. Quality Control solutions (QCs) were prepared at two concentration levels (4 µg/mL and 20 µg/mL) and were derived from a separate stock solution (at 200 mg/mL). The IS was prepared as a working solution of 4 µg/mL in H_2O. All stock standard solutions and QCs were stored at -20°C.

Brain tissue homogenates

Brain tissue from untreated rats (Sprague-Dawley rats, Tg(SNCA)BAC) was used as blank matrix to prepare the calibration curve and QC samples. Different brain regions were snap-frozen in dry ice immediately after dissection and stored at -80 ^{o}C. Tissues were then homogenized using an IKA Ultra Turrax Homogenizer T8 in a tissue-to-lysis buffer ratio of 1:2 (w/v), using the Clarity OTX loading-lysis buffer. On the day of analysis, tissue homogenates were thawed, and 10 µL of sample were spiked with 10 µL of calibration solution (0.5-1-2-4-5-10-20-40-80 µg/mL) or QC solution (4 µg/mL and 20 µg/mL), resulting in calibration samples with concentrations of 1-2-4-8-10-20-40-80-160 ng/mg of tissue and QC samples with concentration of 8 ng/mg and 40 ng/mg, respectively. IS was added to all samples (10 µL of a 4 µg/mL solution). For unknown samples, 10 µL of tissue homogenate were used, 10 µL of IS (4 µg/mL) were added, and 10 µL of H_2O to adjust equal volume as in calibration or QC samples.

Proteinase K digestion

A volume of 10 µL of tissue homogenate was transferred in LoBind tubes, and 8 µL of digestion buffer (15 mM Tris-HCl, 25 mM EDTA, 100 mM guanidine hydrochloride, 0.1% Triton-X 20 mM TCEP) were added along with 6 µL of Proteinase K solution (50 mg/mL in H_2O). The samples were then incubated at 55 ^{o}C for 3 h.

Solid Phase Extraction

Solid Phase Extraction (SPE) of ASO 3.0 was performed using Clarity OTX extraction plates, following the procedure described (Phenomenex, Aschaffenburg, Germany) slightly modified to obtain optimum results. After Proteinase K digestion, samples were left for 10 min at room temperature and then diluted 1/20 in the Clarity OTX Lysis-Loading buffer.

The SPE plate was conditioned with 1 mL of MeOH and 1 mL of equilibration buffer (50 mM ammonium acetate, pH=5,5), and 200 µL of samples in the Loading-Lysis buffer were loaded onto the plate. Samples were washed three times with 1 mL washing buffer (equilibration buffer/ ACN, 1/1). Finally, the analytes were eluted with 1 mL elution buffer (100 mM ammonium bicarbonate, 1 mM TCEP, pH=9.5/ ACN/ THF, 5/4/1) added in two steps of 0.5 mL each. After elution, the samples were transferred into LoBind tubes and evaporated at 55 ^{o}C for approximately 3 h and stored at -20 ^{o}C until analysis.
